# Analysis on urban densification dynamics and future modes in southeastern Wisconsin, USA

**DOI:** 10.1371/journal.pone.0211964

**Published:** 2019-03-06

**Authors:** Lingzhi Wang, Hichem Omrani, Zhao Zhao, Dante Francomano, Ke Li, Bryan Pijanowski

**Affiliations:** 1 College of Earth Sciences, Jilin University, Changchun, Jilin, China; 2 Institute of Geographic Sciences and Natural Resources Research, Chinese Academy of Sciences, Beijing, China; 3 Department of Forestry and Natural Resources, Purdue University, West Lafayette, Indiana, United States of America; 4 Urban Development and Mobility Department, Luxembourg Institute of Socio-Economic Research (LISER), Esch-sur-Alzette, Luxembourg; 5 School of Electronic and Optical Engineering, Nanjing University of Science and Technology, Nanjing, China; 6 Key Laboratory of Songliao Aquatic Environment, Ministry of Education, Jilin Jianzhu University, Changchun, Jilin, China; University of Wisconsin Milwaukee, UNITED STATES

## Abstract

Urban change (urbanization) has dominated land change science for several decades. However, few studies have focused on what many scholars call the urban densification process (i.e., urban intensity expansion) despite its importance to both planning and subsequent impacts to the environment and local economies. This paper documents past urban densification patterns and uses this information to predict future densification trends in southeastern Wisconsin (SEWI) by using a rich dataset from the United States and by adapting the well-known Land Transformation Model (LTM) for this purpose. Urban densification is a significant and progressive process that often accompanies urbanization more generally. The increasing proportion of lower density areas, rather than higher density areas, was the main characteristic of the urban densification in SEWI from 2001 to 2011. We believe that improving urban land use efficiency to maintain rational densification are effective means toward a sustainable urban landscape. Multiple goodness-of-fit metrics demonstrated that the reconfigured LTM performed relatively well to simulate urban densification patterns in 2006 and 2011, enabling us to forecast densification to 2016 and 2021. The predicted future urban densification patterns are likely to be characterized by higher densities continue to increase at the expense of lower densities. We argue that detailed categories of urban density and specific relevant predictor variables are indispensable for densification prediction. Our study provides researchers working in land change science with important insights into urban densification process modeling. The outcome of this model can help planners to identify the current trajectory of urban development, enabling them to take informed action to promote planning objectives, which could benefit sustainable urbanization definitely.

## Introduction

Due to significant population and economic growth, urbanization has occurred around the world at unprecedented rates in recent decades [1–4]. In response to these trends, UN-Habitat has identified planned city infill, redevelopment and densification as three critical areas to focus its global urban development agenda. According to the research of UN-Habitat, most cities in the world have forfeited agglomeration benefits and instead have generated sprawl, congestion and fragmentation over the last two decades [5, 6]. The unstructured nature of urbanization presents great difficulties for developing prudent land use policies by city planning offices [7–11]. Unplanned urban expansion and increased densification may cause a series of environmental and socioeconomic issues such as environmental degradation, loss of agricultural and natural land resources, and shortage or unequal distribution of water resources and associated infrastructure [12–19]. Urban planning should optimize the use of urban land to promote the sustainability of the urban landscape [20]. However, inefficient cities with obsolete urban patterns should be guided by rules that improve densification processes, while undesirable effects, such as gentrification or unreasonable increases in land prices in degraded areas, be restrained (https://unhabitat.org/un-habitat-hosts-global-meeting-on-planning-compact-cities/). Thus, it is essential for urban planners and land use policy makers to actively manage expansion and densification simultaneously [21, 22].

According to the National Land Cover Database (NLCD) of North America [23], developed covers (i.e., urban areas) are placed into 4 broad classes: open space, low-intensity urban, medium-intensity urban, and high-intensity urban [24–28]. Essentially, these classes are defined by percentage impervious surface. These included *developed*, *open space-areas*, which are defined as a mixture of some constructed materials, but mostly vegetation in the form of lawn grasses. Impervious surfaces account for less than 20% of total cover. These areas most commonly include large-lot single-family housing units, parks, golf courses, and vegetation planted in developed settings for recreation, erosion control, or aesthetic purposes. *Developed*, *low intensity-areas* are those with a mixture of some constructed materials and vegetation. Impervious surfaces account for 20–49% of total cover. These areas most commonly include single-family housing units. *Developed*, *medium intensity-areas* are locations with a mixture of some constructed materials and vegetation. Impervious surfaces account for 50–79% of total cover. These areas most commonly include single-family housing units. Finally, *developed*, *high intensity-high developed areas* are where people reside or work in high numbers. Examples include apartment complexes, row houses and commercial/industrial. Impervious surfaces account for 80–100% of total cover), we treated them as *open space* (OS), *low density* (LD), *medium density* (MD), and *high density* (HD) to express the urban intensities represented in this paper [29–31].

Southeastern Wisconsin (SEWI), USA has undergone striking urbanization in the past 3 decades [32]. According to 10 years of NLCD change data, the urban footprint of SEWI has changed considerably from 2001 to 2011. Considering the county of Milwaukee—the urban and economic center of SEWI—a spatial analysis of these two time periods shows that commercial, industrial and recreational areas increased, 48%, 45%, and 50%, respectively, in size. As these might reflect different urban densities, have densities of urban changed as well over time? What are the relationships between these broad urban classes and urban densities?

Broadly, managing densification as a planning strategy, which can be considered an effective tool for improving sustainability of cities, has gained much attention in the public but little in the area of research [33, 34]. Urban planning offices have used forecast models to examine sustainable futures [35] but none have been developed to address densification. Taking into account the current and possible future urban densities using simulations could enhance the accuracy and timeliness of urban land planning in places such as SEWI.

A wide variety of land use models have been developed to simulate urbanization, and a diverse set of tools have been applied. For example, cellular automata (CA), which predicts urban expansion based on specified or learned neighborhood functions [36–41], have been extremely popular. One of the most widely used CA-based model is SLEUTH (Slope, Land use, Excluded, Urban, Transportation, Hill shade), which forecasts urban development based on a core urban growth model (UGM) and the deltatron land use/land cover model (DLM; [39, 42–44]). The Land Transformation Model (LTM), which developed nearly 20 years ago, applies machine-learning capabilities of artificial neural networks, has been applied to a variety of locations including the Midwestern USA, Central Europe, East Africa, and Asia [45–47]. It has been used to simulate land cover changes and to predict urban boundary changes [32, 48–54]. However, most studies using the LTM have not addressed differences in urban density nor the process of densification. The LTM, likes other models, treats urban cells as a single layer or focuses only on transitions (for instance from non-urban to urban areas) and ignores intensity levels, which plays a vital role in defining the quality of urban [49, 53, 55]. Incorporating such feature into the LTM could allow researcher and policy analysts to begin to study urban densification with a well-known land change-modeling tool.

Urban change (urbanization) has dominated land change science work for the last several decades. However, there are very few studies on urban densification process (urban intensity expansion) despite its importance. To fill this gap, the present study used a rich dataset for the contiguous USA and adapted the well-known land transformation model (LTM) to explore this topic [56]. Here, we reconfigured the LTM to examine urban densification based on changes of urban land densities in SEWI which were then used to project to the future.

The objectives of this paper are to: 1) quantify the past (2001–2011) transition process between different densities of urban land in SEWI, 2) develop a method to simulate multiple urban densities using a reconfigured LTM; 3) predict future urban densification in SEWI that could guide urban land management, and 4) present our lessons to be learned from urban densification in SEWI.

## Study area and dataset

### Study area

**Fig 1:**
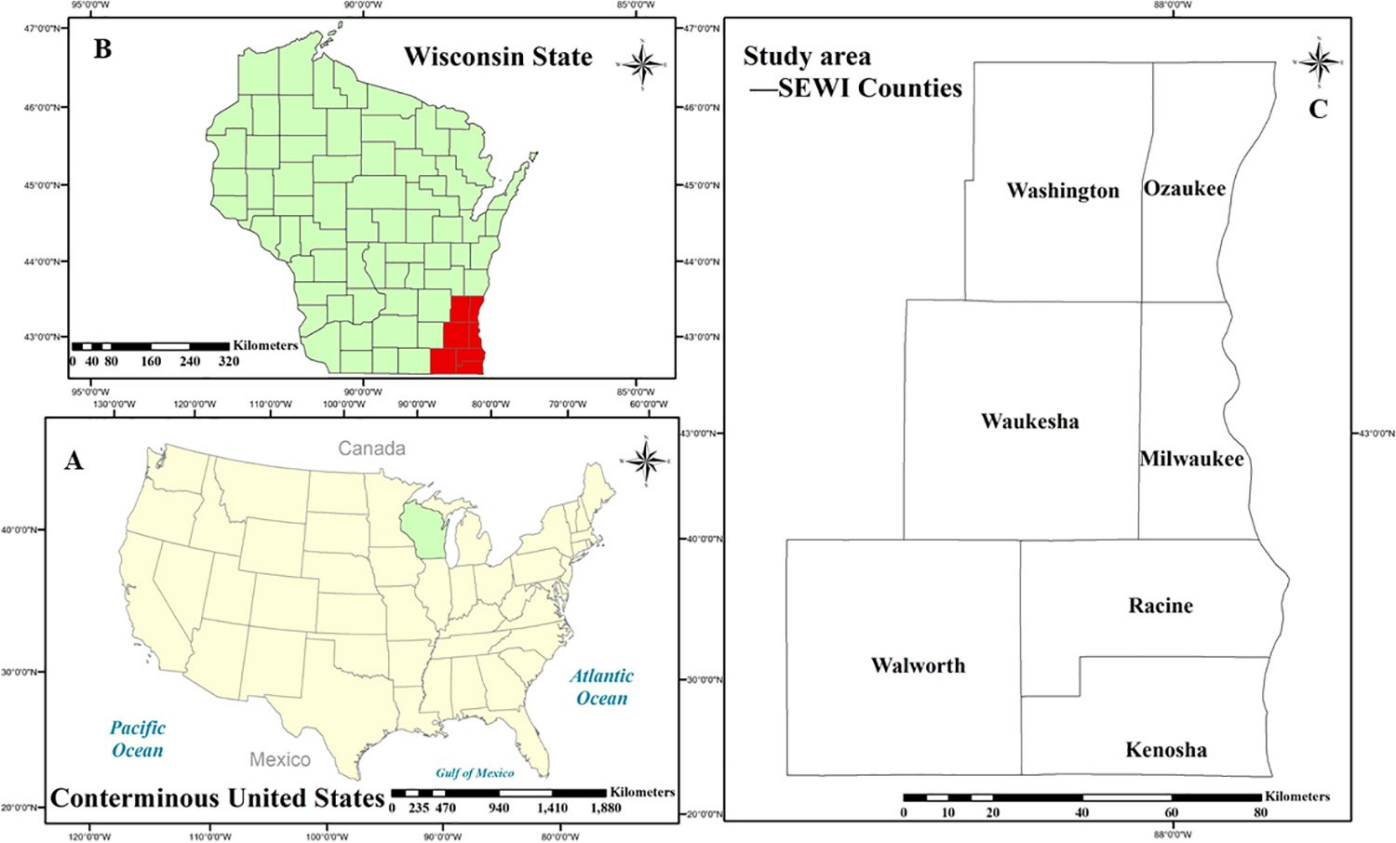
Location of southeastern Wisconsin (SEWI). (A) Wisconsin State in Conterminous United States.(B) Southeastern Wisconsin in Wisconsin State.(C) Counties in southeastern Wisconsin.

Wisconsin is a state located in the north-central United States. Southeastern Wisconsin (SEWI) comprises seven counties: Kenosha, Milwaukee, Ozaukee, Racine, Walworth, Washington, and Waukesha Counties [8, 32] (as shown in Fig 1). SEWI is currently dominated by agriculture, urban, and forest, which accounted for more than 86% of the landscape in 2011 (47.33%, 26.92%, and 12.08% respectively; Table 1). Between 2001 and 2011, the percentage of urban increased from 24.37% to 26.92%, whereas agriculture and forest decreased by 2.09% and 0.31%, respectively. More than 60% of lost agriculture contributed to urban gain during the 10-year period. SEWI has undergone remarkable urbanization between 2001 and 2011.

**Table 1:** Percent of land use classes of SEWI in 2001, 2006, and 2011 and change in percent coverage from 2001 to 2011.

Land use classes		2001	2006	2011	2011–2001
Agriculture	Pasture/hay	11.40	11.11	10.96	-0.44
	Cultivated crops	38.02	36.89	36.36	-1.66
	*Total agriculture*	*49*.*42*	*48*.*00*	*47*.*33*	*-2*.*09*
Forest	Deciduous forest	11.39	11.11	11.10	-0.29
	Evergreen forest	0.22	0.22	0.23	0.00
	Mixed forest	0.77	0.76	0.76	-0.02
	*Total forest*	*12*.*39*	*12*.*10*	*12*.*08*	*-0*.*31*
Urban	Open space	8.10	8.89	9.14	1.04
	Low density	9.74	10.12	10.31	0.57
	Medium density	4.77	5.16	5.39	0.62
	High density	1.76	1.96	2.08	0.31
	*Total urban*	*24*.*37*	*26*.*13*	*26*.*92*	*2*.*54*
Other classes	Total other classes	13.82	13.78	13.67	-0.15

### Dataset

The classification system used by the NLCD is modified from the Anderson Land Cover Classification System [23]. NLCD 2001, 2006 and 2011 land use/cover databases are based primarily on a hierarchical classification system developed in the late 1970s from Landsat satellite data [57–59]. According to the NLCD classification system, there were 8 subclasses in level 1 and 15 subclasses in level 2 in SEWI. ArcGIS was used to generate 2001, 2006, and 2011 maps that aggregated these 15 classes down to 5: *non-urban* (NU), *open space* (OS), *low density* (LD), *medium density* (MD), and *high density* (HD). While both urban expansion and urban densification occurred during 2001–2006 and 2006–2011, in this study we primarily consider urban densification, which includes transitions from OS to LD, MD, and HD; LD to MD and HD; and, MD to HD. Predictor variables derived from the NLCD data included the class of each cell itself, the distance to the nearest OS, LD, MD, and HD cell, and the density of OS, LD, MD, and HD around each cell.

Sixteen spatial predictor variables were used to evaluate urban densification (Table 2) generated with ArcGIS 10.3 and based on: i) NLCD data from 2001, 2006, and 2011, ii) a digital elevation model (DEM) from the U.S. Geological Survey (USGS), and iii) road, stream, and park data extracted from land use maps. These sixteen spatial predictor variables are ones common to land change modeling (see [60] for review of concepts) found in ANN, multiple regression and logit models where a host of a dozen or so independent variables are used to predict one or more dependent variable (change/no change of a use). Variables 9–16 have traditionally been represented as one predictor variable (distance to urban) but as we would like to test the notion that there are strong relationships between predictor variables and urban intensity, we split these out into subclasses of urban density. All data were in raster file format stored and processed at a resolution of 30 meters.

**Table 2:** Spatial predictor variables used in this study.

Items	Predictor variables	Abbreviation	Type
1	Elevation	Elevation	Numeric
2	Slope	Slope	Numeric
3	Distance to road	Dis_Road	Numeric
4	Distance to stream	Dis_Stream	Numeric
5	Distance to park	Dis_Park	Numeric
6	Distance to water	Dis_Water	Numeric
7	Distance to open space	Dis_OS	Numeric
8	Density of open space	Den_OS	Numeric
9	Distance to low density	Dis_LD	Numeric
10	Density of low density	Den_LD	Numeric
11	Distance to medium density	Dis_MD	Numeric
12	Density of medium density	Den_MD	Numeric
13	Distance to high density	Dis_HD	Numeric
14	Density of high density	Den_HD	Numeric
15	Distance to non-urban	Dis_NU	Numeric
16	Density of non-urban	Den_NU	Numeric

The rationale for using each predictor variable is as follows. First, elevation and slope are the natural foundation for urban densification[61]. We obtained elevation, and slope variables from the DEM. Slope was calculated using the Spatial Analyst tool in ArcGIS. Second, since access to different urban density affects urban density development patterns, the distance variables are expected that sites nearer to existing urban density would be more likely to develop to the same densities[44, 62, 63].The minimum Euclidean distance to each feature of urban densification (e.g. open space, low density, medium density and so on) was calculated in ArcGIS10.3. The density variables represented the amount of different urban density, indicated the degree to which the urban was dominated by different urban densities. The neighborhood function in ArcGIS was used to calculate the density of each main urban density class around the focal cell (e.g., 1.2 km) [63]. Third, the accessibility to transportation provided accessibility that influences speed and direction of spatial densification growth [64, 65]. Distance to streams and water were the predictors related to service supply (water resources), while distance to park indicated the distance from recreational site and also urban landscape quality among different urban densities[33].

## Methods

### Analysis of past urban densification

We used land use maps and ArcGIS to generate a land use transition (2001–2011) matrix and to explore the temporal and spatial changes of urban density for SEWI. In this study, several landscape metrics were employed to describe changes in the spatial patterns of urban categories and to assess the nature of model errors (Table 3) [66–69]. Ring-based analysis, which is firmly grounded in classical urban theory, was also used to reveal the transition characteristics of urban densification within the county of Milwaukee. The county of Milwaukee is the urban and economic center of SEWI, which was the “focusing” place of high density and medium density areas and presented the most typical urban densification of SEWI. The center of this ring-based analysis was defined as the historical center of the city of Milwaukee, which is located approximately at 43°03′35.949” N, 87°48′39.347” W. Multiple ring buffers were created at 1 km intervals around this center from 1 km to 10 km using ArcGIS. In this ring-based analysis, we considered percentage of OS, LD, MD, and HD, as well as the Urban Expansion Rate (UER), using the following equation:

$${\mathrm{UER}}_{i,t∼t+n}=\frac{{\mathrm{UD}}_{i,t+n}‑{\mathrm{UD}}_{i,t}}{{\mathrm{UD}}_{i,t}}\times \frac{1}{n}\times 100$$ (1)

where *UER*_*i*,*t~t+n*_ is the urban expansion rate of the *i*^th^ buffer ring, *UD*_*i*,*t+n*_ and *UD*_*i*,*t*_ are the urban density areas of year t+n and year t, respectively [70–73].

**Table 3:** Description of landscape metrics used in this study.

Abbreviation	Metrics	Description	Units	Range
Number of Patches	NP	(Class) NP equals the number of patches of the corresponding patch type (class). (Landscape) NP equals the number of patches in the landscape. Note, NP does not include any internal background patches (i.e., within the landscape boundary) or any patches at all in the landscape border, if present.	N/A	NP≥1, no limit
Landscape Shape Index	LSI	LSI equals .25 (adjustment for raster format) times the sum of the entire landscape boundary (regardless of whether it represents 'true' edge or not, or how the user specifies how to handle boundary/background) and all edge segments (m) within the landscape boundary (Class: involving the corresponding patch type), including some or all of those bordering background (based on user specifications), divided by the square root of the total landscape area (m2). Note, total landscape area (A) includes any internal background present.	N/A	LSI≥1, no limit
Contagion Index	CONTAG	(Landscape) CONTAG equals minus the sum of the proportional abundance of each patch type multiplied by the proportion of adjacencies between cells of that patch type and another patch type, multiplied by the logarithm of the same quantity, summed over each unique adjacency type and each patch type; divided by 2 times the logarithm of the number of patch types; multiplied by 100 (to convert to a percentage). Note, Pi is based on the total landscape area (A) excluding any internal background present.	Percent	0 < CONTAG ≦ 100
Largest Patch Index	LPI	(Class) LPI equals the area (m^2^) of the largest patch of the corresponding patch type divided by total landscape area (m^2^), multiplied by 100 (to convert to a percentage). Note, total landscape area (A) includes any internal background present.	Percent	0<LPI≤100
Edge Density	ED	(Class) ED equals the sum of the lengths (m) of all edge segments involving the corresponding patch type, divided by the total landscape area (m^2^), multiplied by 10,000 (to convert to hectares). Note, total landscape area (A) includes any internal background present.	Meters per hectare	ED≥0, no limit
Fractal Dimension Index	FRAC	(Class) FRAC equals 2 times the logarithm of patch perimeter (m) divided by the logarithm of patch area (m^2^); the perimeter is adjusted to correct for the raster bias in perimeter.	N/A	1≤FRAC≤2
Contiguity Index	CONTIG	(Class) CONTIG equals the average contiguity value (see discussion) for the cells in a patch (i.e., sum of the cell values divided by the total number of pixels in the patch) minus 1, divided by the sum of the template values (13 in this case) minus 1.	N/A	0≤CONTIG≤1

### Land transformation model

**Fig 2:**
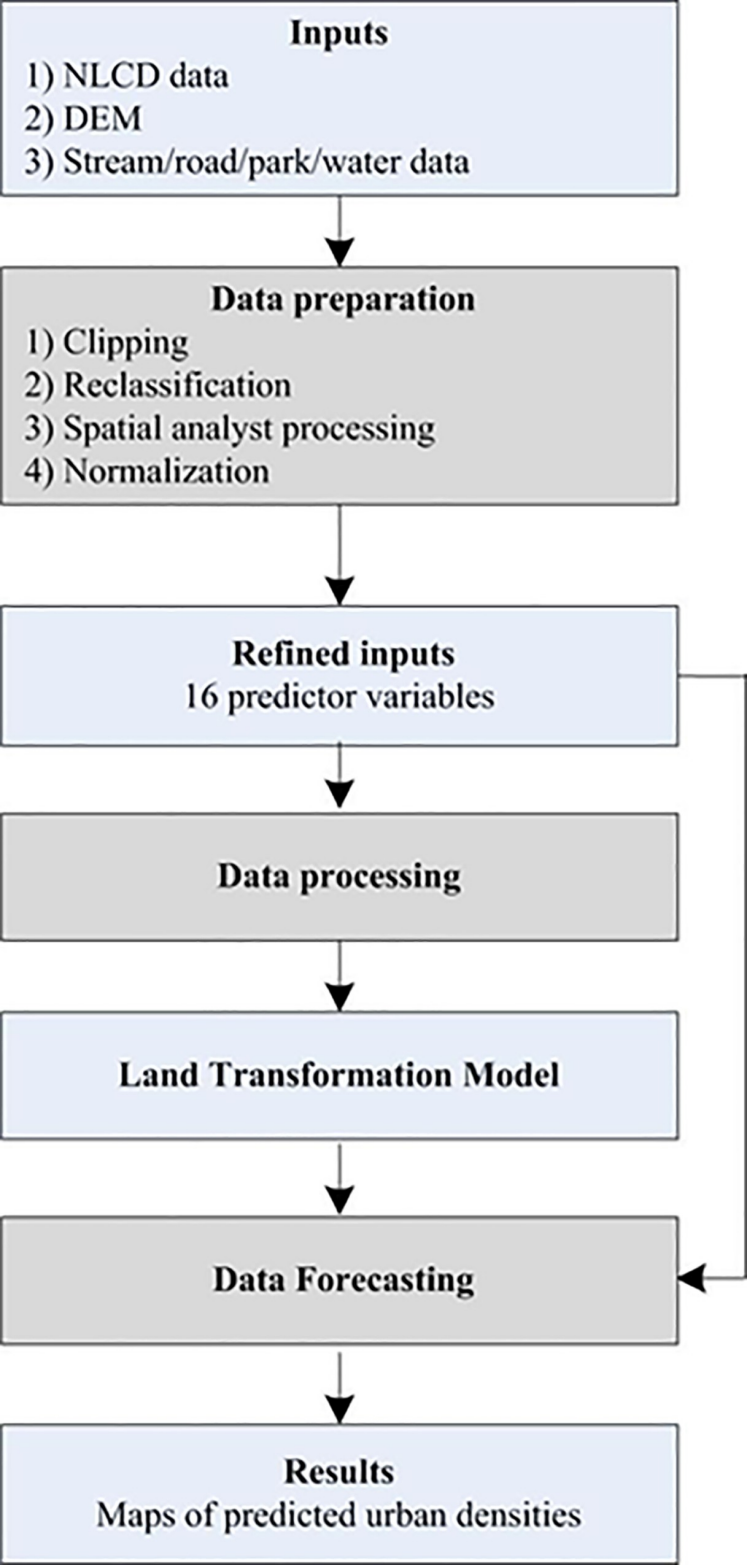
Modeling steps of the LTM.

The LTM, which couples GIS with artificial neural networks (ANNs) to simulate land use change, utilizes a raster modeling environment to simulate urban growth based on a variety of socio-economic and bio-physical factors (for details, see: [45, 48, 54]). Based on historical land use change data and predictor variables, the ANN (hereafter as neural network) learns patterns of urban densification; this information is then saved and used to forecast change (i.e., future urban densification) (Fig 2). LTM modeling follows three steps: 1) Data preparation: the predictor variables were created. 2) Data processing: the spatial transition rules governing urban density transitions were learned. 3) Forecasting: the urban density changes based on the calibrated model were simulated. In Step 1, the inputs included the urban density classes and the spatial drivers influencing urban density changes such as the distance to urban density areas and density of urban areas. All the inputs are often normalized as they are presented to the neural network. In Step 2, an ANN algorithm was applied to mimic the urban densification processes based on the predictor variables. In Step 3, the calibrated model (from Step 2) was used to simulate future urban densification. For example, one may use drivers in 2001, 2006, 2011, or 2016 to predict urban density in 2006, 2011, 2016, or 2021, respectively.

### Model calibration and validation

Unbiased interpretation of model performance was a significant part of modeling. As there was not a single calibration metric for land use models that can provide an unbiased outcome [32, 74–76], we used multiple goodness-of-fit metrics to evaluate the performance of the model and produced an unbiased outcome for this study [49, 77]. First, we created confusion matrices of observed and simulated urban density classes for 2006 and 2011. Next, we compared all urban density classes between these two maps (i.e. observed versus simulated maps for each year) in each cell to quantify error locations [78–80]. We also used the Receiver Operating Characteristic (ROC) and the Area Under the ROC Curve (AUC) to quantify the goodness-of-fit of the LTM model[63]. The ROC curve visually depicted model accuracy across a range of thresholds (between 0 and 1), which generated x- and y-axes (false positive or FP and true positive or TP rates as a function of threshold values) to plot the ROC curve. The outcome of the model was a set of probabilities between 0 and 1. These probabilities were compared to thresholds to decide the class membership and then to compute the TP and FP rates. The AUC provided the accuracy of the model with a real number between 0 and 1. Larger values of AUC corresponded to better performance. We then compared spatial patterns of urban land patches between observed and simulated maps for 2006 and 2011. Finally, we created error maps that illustrate the size, configuration, and location of all simulation errors. All these metrics were used in the next section.

## Results

### Analysis of urban densification dynamics

#### Urban densification dynamics of SEWI

**Fig 3:**
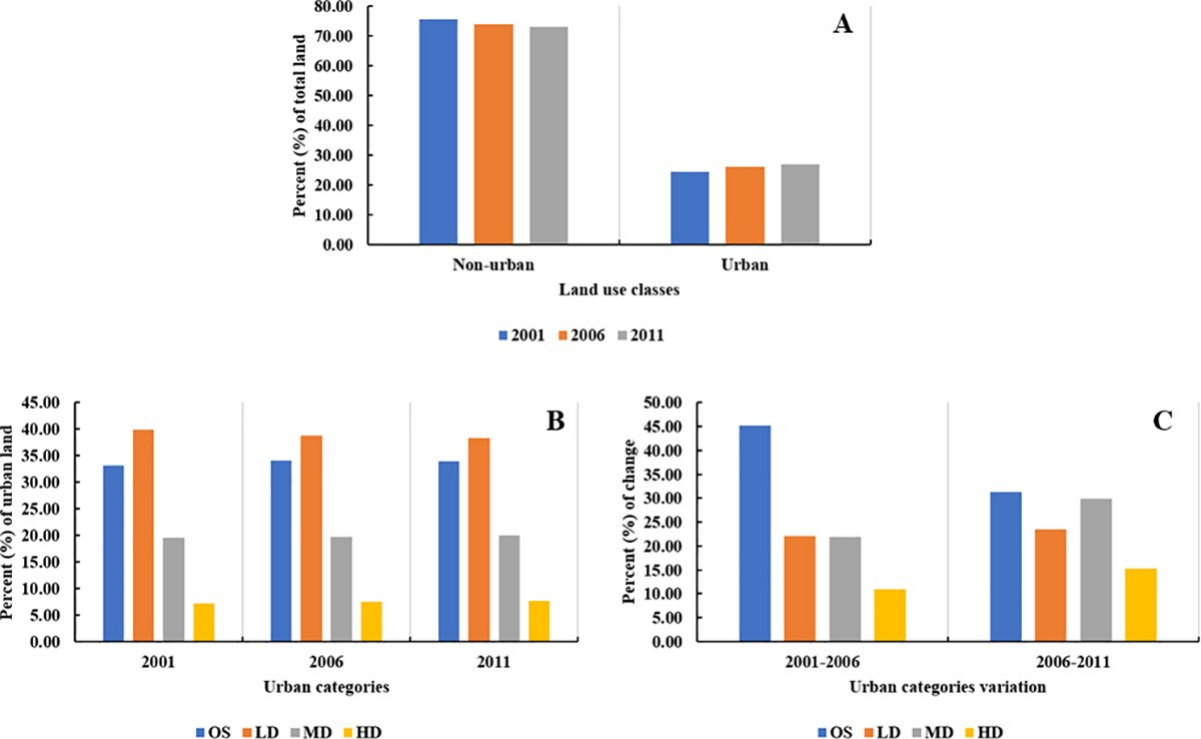
Urbanization dynamics between 2001 and 2011. (A) Non-urban and urban variation (percent of total land) in 2001, 2006 and 2011.(B) Urban categories variation (percent of urban land) in 2001, 2006 and 2011.(C) Urban categories variation (percent of change) between 2001 and 2011.

**Fig 4:**
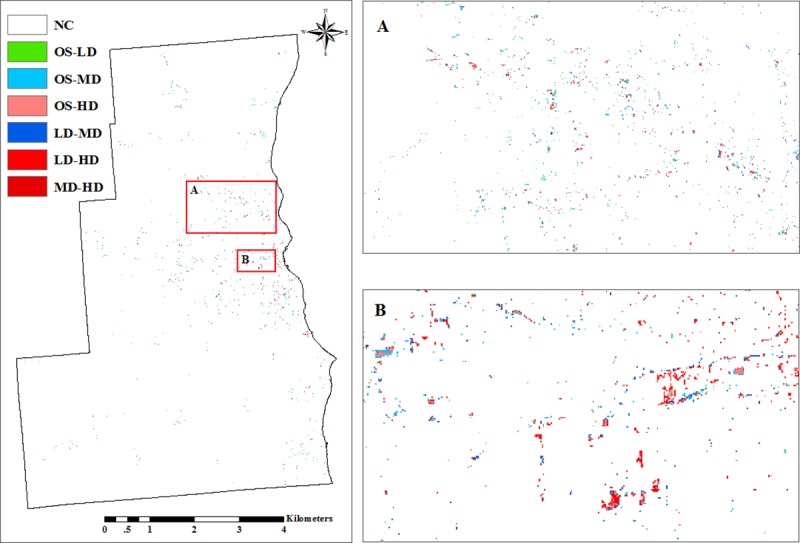
Urban density transition maps between 2001 and 2011. (A) Urban density transition far from the historical center. (B) Urban density transition near the historical center.NC means no change, and labels of the form XX-YY indicated that XX transitioned to YY.

We found that land use change in SEWI between 2001 and 2011 was generally characterized by a decrease in non-urban area and an increase in urban area. Fig 3 showed the increase in urban area with growth rates of 7.21% between 2001 and 2006, 3.01% between 2006 and 2011, and 10.44% overall between 2001 and 2011. Regarding urban density classes (and including transitions from NU), open space increased most substantially by 40.89% for the entire 10-year period. Low-density and medium-density areas increased by 22.50% and 24.33%, respectively, whereas high-density areas increased the least (12.28%; Fig 4). These universal increases indicated that densification was an important and indispensable process that was as important as urbanization. Considering the increasing proportion of the density, we found that while all urban densities in SEWI were increasing relative to total County area, the overall character of urban areas in 2011 was lower-density as compared to 2001.The non-urban area transferred to open space along with the urbanization was one possible reason for the increasing proportion of lower-density.

The lower density may account for the increasing proportion of lower density in SEWI from 2001 to 2011 according to the densification transitions. There was no higher density transfer to lower density between 2001 and 2011, the predominant form of densification were transitions from open space areas to higher-density areas (0.89% of the original open space lost) followed by transitions from low density to medium and high density (0.59% of the original lost) and transitions from medium to high density (0.05%; of the original lost Table 3). It means that once areas have become urban (open space), they almost exclusively transition to higher density subclasses. As one would expect, densification (as detectable via remote sensing) of higher-density areas become progressively less common, likely because such development poses greater logistical challenges than development of open-space urban areas. The lower density trends illustrate that densification was a progressive process that become asymptotically more difficult to achieve and/or detect (Table 4, Fig 4).

**Table 4:** Urban density transition matrix in SEWI (2001–2011 by percent transitioned).

	Urban densities in 2001						
Urban densities in 2011		OS	LD	MD	HD	Total gain	Total
	OS	32.34	0.00	0.00	0.00	0.00	32.34
	LD	0.14	39.36	0.00	0.00	0.14	39.50
	MD	0.58	0.26	19.53	0.00	0.83	20.37
	HD	0.18	0.33	0.05	7.24	0.56	7.79
	Total lost	0.89	0.59	0.05	0.00	1.53	——
	Total	33.23	39.95	19.58	7.24	——	100.00

Urban densification proceeds in an unstructured manner, and the spatial pattern of urban categories changed substantially between 2001 and 2011 (Table 5). The increasing ED and LSI of MD (ED: 6.48; LSI: 22.85) indicates increased fragmentation and complexity of medium-density areas. The increase of PARA_MN of HD suggests that high-density areas acquire more complex shapes than they had in 2001 (24.36).

**Table 5:** Changes of Landscape metrics of urban densities in SEWI (2001–2011).

TYPE	NP	LPI	ED	LSI	PARA_MN	CONTIG_MN
OS	3,508	-0.02	5.75	20.21	-10.72	0.01
LD	4,992	-0.10	3.48	17.16	5.84	0.00
MD	4,544	-0.24	6.48	22.85	-0.94	0.00
HD	2,842	0.21	3.48	14.88	24.36	-0.02

In summary, urban densification was an important and progressive process along with the urbanization. The increasing proportion of lower density areas rather than higher density areas was the main characteristic of the urban densification in SEWI from 2001 to 2011. Construction on non-urban areas and open space areas means lower development cost including lower land price, less original urban architecture and more “free” expand space than on the higher density areas. However, these “freestyle” urban expansion modes were prone to causing disorderly urban sprawl and wasted development potential across the metropolitan area. Controlling the city size and improving the urban land use efficiency are effective pathways for sustainable urbanization.

#### Urban densification dynamics of a sample county—Milwaukee

**Fig 5:**
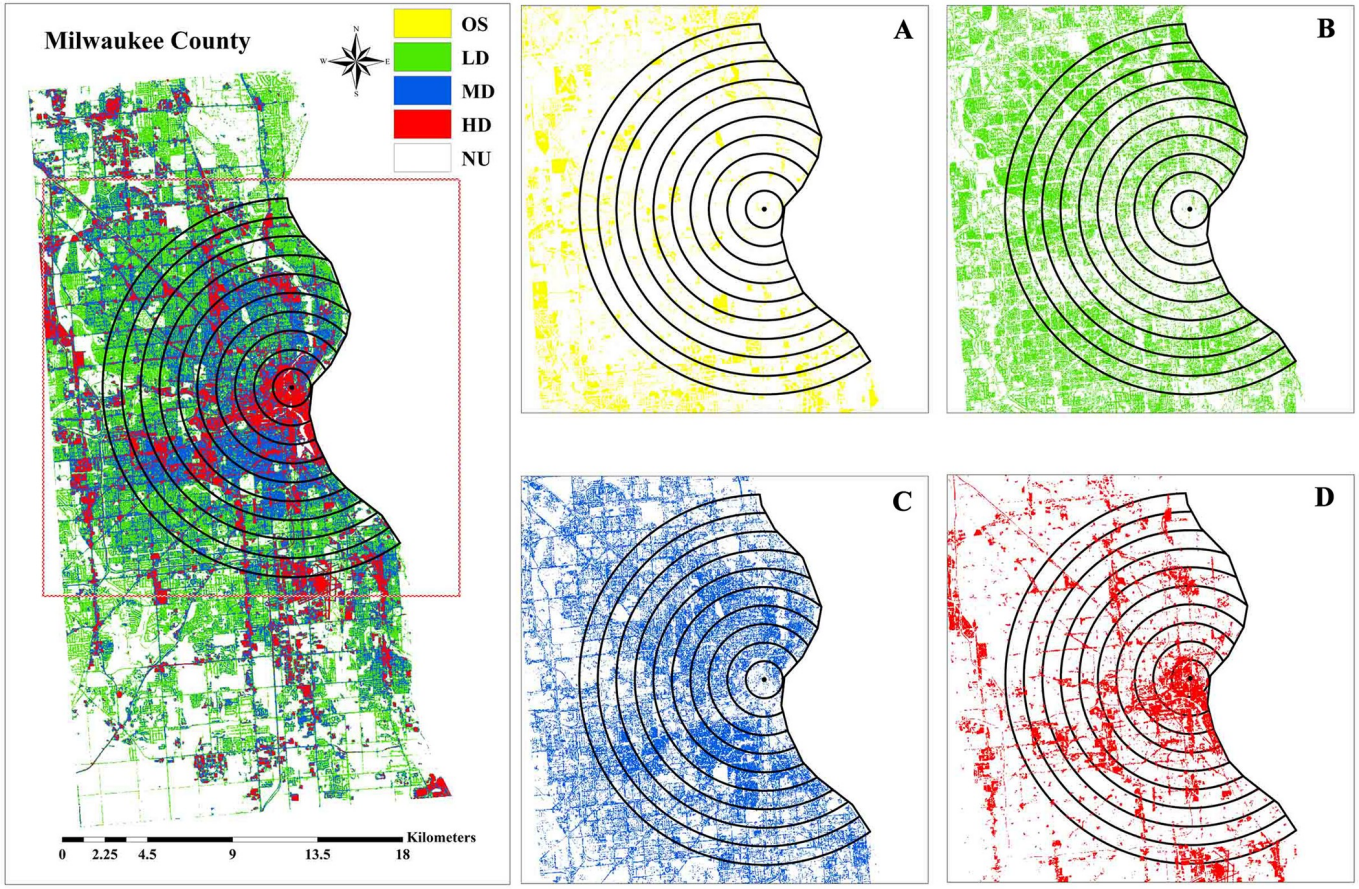
Urban density classes within 1–10 km buffers in Milwaukee County in 2011. (A) Open-space areas variation within 1–10 km buffers. (B) Low-density areas variation within 1–10 km buffers. (C) Medium-density areas variation within 1–10 km buffers. (D) High-density areas variation within 1–10 km buffers. NU represented non-urban areas.

The county of Milwaukee is the economic and geographic center of SEWI. The proportion of medium and high density areas of the Milwaukee County nearly accounted for 50% (40.59% & 46.27%) compared to 50% of all the other six counties. The urban densification of Milwaukee County could describe the densification characteristic of SEWI more clearly and more directly. To consider spatial variability in urban density and urban densification, we created 10 ring buffers at 1 km intervals around the historical center of the City of Milwaukee in Milwaukee County (Fig 5).

We calculated the percentage of urban land use for OS, LD, MD and HD in 2011 and UER (2001–2011) for each buffer ring. By examining the variations in urban density and densification in relation to distance from the urban center, we can characterize how this spatial variable accounts for variation of urban density and densification.

**Fig 6:**
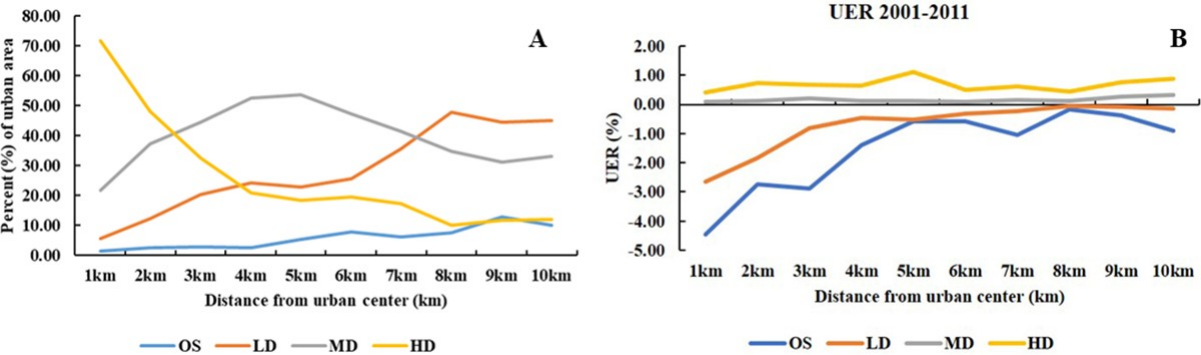
Urban density classes and Urban Expansion Rate (UER) variation at varying distances from the urban center of Milwaukee County. (A) Urban density classes variation within 1–10 km buffers. (B) Urban Expansion Rate (UER) variation within 1–10 km buffers.

From the geographic center outwards in 2011 (Fig 6), the proportion of high-density areas sharply decreased monotonically from 71.68% at 1 km to 20.82% at 4 km and then flattened from 4–7 km (average value: 18.91%) and 8–10 km (average value: 11.17%). High-density areas decreased greatly from the urban core to inner urban areas and then more slowly in outer buffers. Medium-density areas increased within 5 km of the urban center (from 21.56% to 53.65% between 1 and 5 km) and then decreased from 47.21% to 31.24% between 6 and 9 km. Open space areas increased gradually, and low-density areas increased greatly from the urban center with especially sharp increases between 1 and 4 km (1.38% to 2.51%) and between 5 and 8 km (5.24% to 7.52%). This density variation was consistent with our current understanding of urban density, and it illustrated that the urban distribution within Milwaukee County was in accordance with typical rules.

The most obvious feature of UER variation between 2001 and 2011 was that the UER of high-density and medium-density areas was consistently positive across space, whereas the UER of open space and low-density areas were consistently negative, and markedly so near the geographic center. High- and medium-density areas expanded and open space and low-density areas shrank substantially during this period (Fig 6). The high density UER was highest at 5 km, and the average between 1 and 4 km (0.62%) was smaller than that of 7–10 km (0.67%), indicated that high density expansion in the urban core was slightly more intensive than in outer urban areas. The increasing UER of open space and low-density areas indicated that high density took the place of open space and low-density areas in the urban core. On account of pressure of population and limited urban land supply in the urban core, higher density area presented a characteristic of intensive expansion and fragmentation instead. Another issue that needed to be addressed along with the densification were the increasing runoff and land fragmentation due to the increasing impervious surfaces, the reducing green space and air quality due to growing residential density. We believed that rational densification is equally important as an urbanization consideration.

### Model validation

#### Quantifying error locations

**Fig 7:**
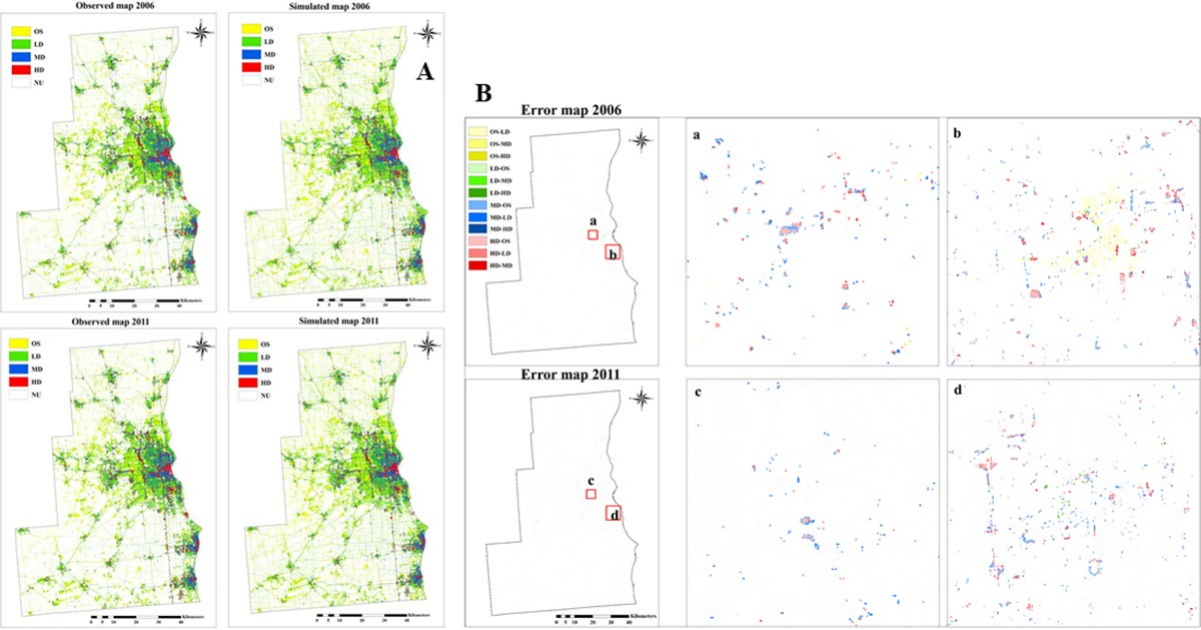
Observed, simulated maps and error maps for 2006 and 2011. (A) Observed, simulated maps for 2006 and 2011.(B) error maps for 2006 and 2011.Labels of XX-YY means XX was observed, but predicted to be YY.

We used observed and simulated maps of 2006 and 2011 to quantify the location/quantity of errors and to produce maps of transition error types for the study area (Fig 7). The simulated maps showed that the model predicted more expansion of open space and low-density areas than was observed. The over-predicted open space and low-density areas occurred in the central portion of the study area (Milwaukee and Waukesha County), which has undergone the most intensive urbanization. Fig 7 provided a clearer illustration of error locations. Transition errors for low density appeared to be scattered throughout the east-central portion of the study area, especially in Milwaukee County.

#### Error quantities for urban density classes

The diagonal values of the confusion matrix (Table 6) summarized the correctly predicted total number and percentage of cells in the maps. The model performed well to simulate most of the land use classes in 2006 and 2011. In general, the simulated maps were very close to the observed maps (Fig 7). However, the diagonal values of the medium and high-density areas were slightly lower than those of the other classes at 97.52% and 95.15%, respectively, in 2006, and 98.41% and 97.73%, respectively, in 2011, which suggested that medium- and high-density areas were more difficult to simulate based on our drivers. This difficulty was likely because the medium- and high-density areas were more concentrated in the urban core, where land use patches were small and complex-shaped, compared to the larger, simpler-shaped open space areas. In general, the model over-predicted open space and low-density areas for both 2006 and 2011. Observed medium-density predicted as open space was the most common error of the model (1.74% and 1.07% in 2006 and 2011, respectively). Another notable trend was that low-density areas were erroneously predicted instead of observed medium-density and high-density areas (0.74% and 2.89%, respectively, for 2006; 0.52% and 1.36%, respectively, for 2011).

**Table 6:** Confusion matrix for 2006 and 2011.

	Simulated map for 2006				
Observed map for 2006		OS	LD	MD	HD
	OS	614,355 (99.76)	2 (0.00)	1,411 (0.23)	42 (0.01)
	LD	1,479 (0.20)	745,938 (99.79)	17 (0.00)	50 (0.01)
	MD	6,590 (1.74)	2,785 (0.74)	368,636 (97.51)	34 (0.01)
	HD	2,159 (1.51)	4,144 (2.89)	646 (0.45)	136,386 (95.15)
	Simulated map for 2011				
Observed map for 2011		OS	LD	MD	HD
	OS	608,400 (99.81)	11 (0.00)	1,136 (0.19)	27 (0.00)
	LD	1,079 (0.14)	742,541 (99.74)	11 (0.00)	862 (0.12)
	MD	4,095 (1.07)	2,005 (0.52)	377,744 (98.41)	20 (0.01)
	HD	1,006 (0.69)	1,992 (1.36)	336 (0.23)	143,409 (97.73)

Note: Percentages are given in parentheses.

**Fig 8:**
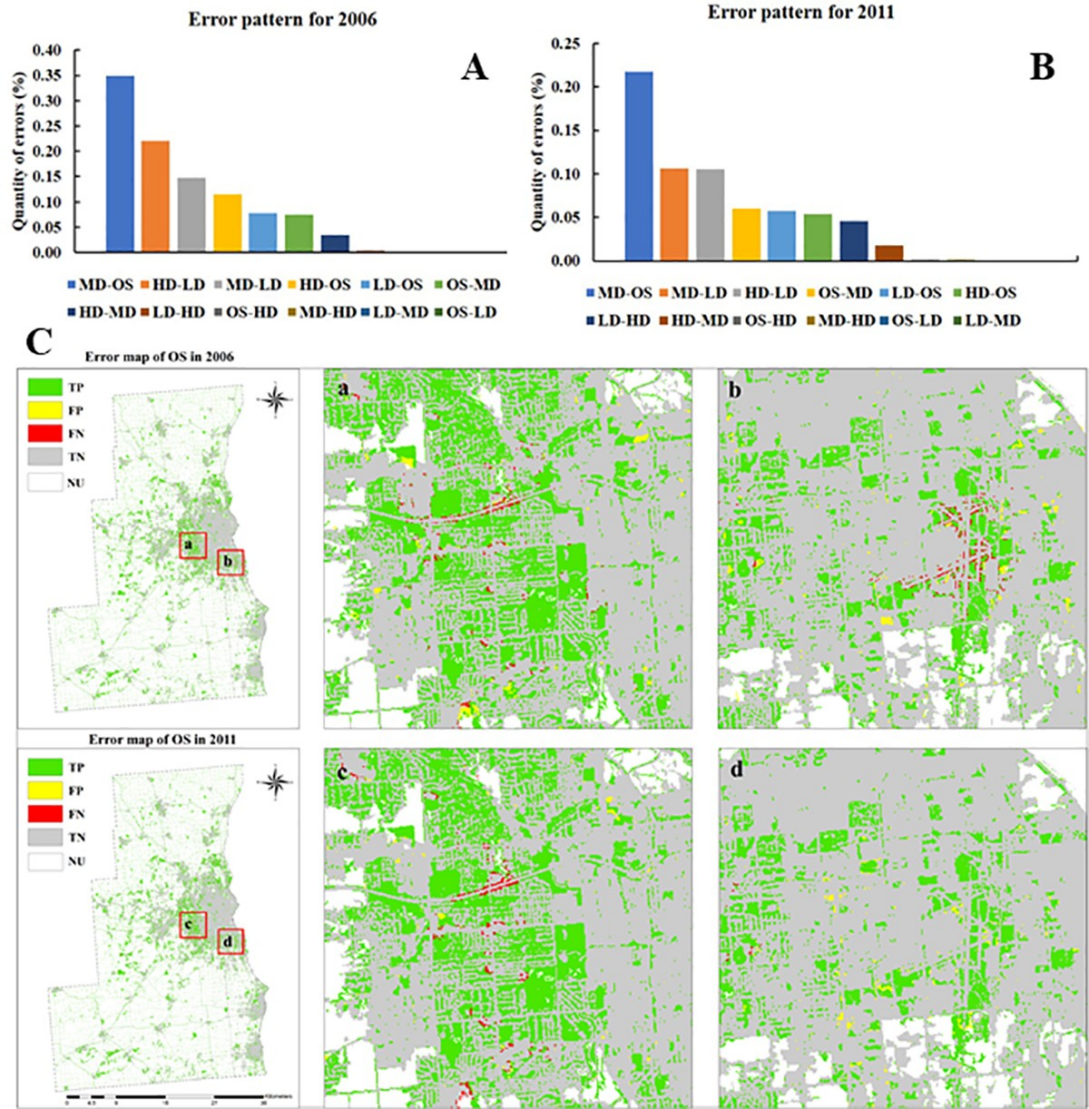
Errors between observed and simulated maps and error maps for open space. (A) Errors between observed and simulated maps as percent of total urban area for 2006. (B) Errors between observed and simulated maps as percent of total urban area for 2011. (C) Error maps for open space for 2006 and 2011.Labels of XX-YY means XX was observed, but predicted to be YY. True positive (TP) represented cells that were predicted to change and did change. False positive (FP) represented cells that were predicted to change but did not change. True negative (TN) represented cells that were predicted to not change and did not change. False negative (FN) represented cells that were predicted to not change but did change. NU represented non-urban areas.

The most abundant error in our model (Fig 8) was observed medium-density areas that were predicted as open space areas. Nearly 0.35% of our 2006 and 0.22% of our 2011 error maps were in this error transition category. According to error maps of predicted open space areas (Fig 8), the model had a high level of accuracy, as shown in green (true positives or TP) and grey (true negatives or TN). False negatives (FN) for open space areas (where the model under-predicted densification) were primarily located along roads and airport runways, whereas false positives (FP; where the model over-predicted densification) were dispersed across the error map.

Table 7 showed the model performance based on the AUC at three levels. First, focusing on the changed cells (i.e. cells with changed states in the entire dataset), the AUC was 0.689. It is important to focus on these types of cells to realistically predict the number of cells that actually change their state within the system and to detect the cells that are more sensitive to change [81–83]. Following this step, the model was allowed to quantify the change potential in the entire land use system (testing and learning sets). Second, we tested the model at the level of cells belonging to the testing set (with both change and non-change), and we found an AUC of 0.977. Here, the testing set was only used for the validation since the learning set is used for the model calibration. Then, the AUC, considering both change and non-change cells, from the entire dataset was 0.980. The high values of AUC illustrated clearly that the model performed satisfactorily for simulating urban density change despite its complexity.

**Table 7:** Area under the ROC curve (AUC) for three validation sets.

Subset	AUC
Changed cells	0.698
Testing set	0.979
Entire set	0.980

#### Landscape metrics of observed and simulated urban class change

**Fig 9:**
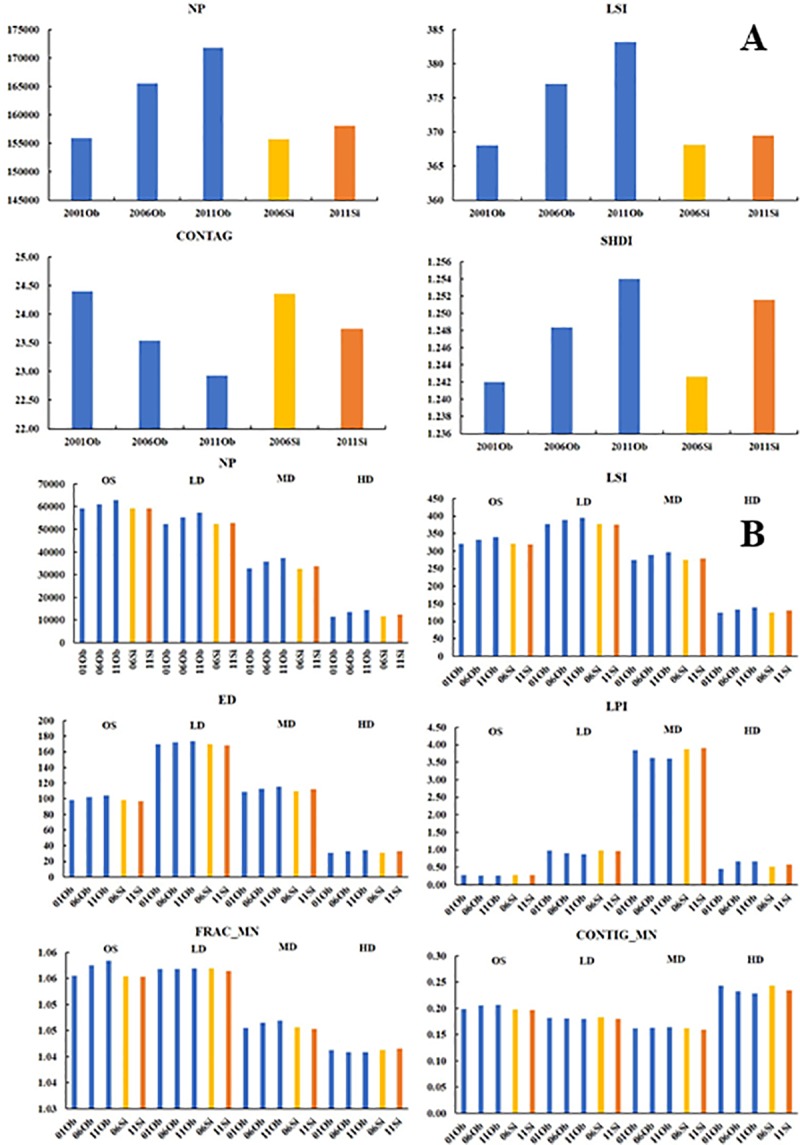
Landscape metrics over time. (A) Landscape metrics in landscape scale in observed (2001, 2006, 2011) and simulated maps (2006, 2011). (B) Landscape metrics in class scale in observed (2001, 2006, 2011) and simulated maps (2006, 2011). “Ob” represented observed map, and “Si” represented simulated map.

A summary of landscape configuration metrics of the simulated maps illustrates some differences with the observed maps (Fig 9). For number of patches, landscape shape indicator, contagion, and Shannon’s diversity index, simulated 2006 was more similar to observed 2001 but was a smaller value than observed 2006 maps. The number of patches and Shannon’s diversity index of simulated 2011 were fewer than observed 2011, whereas the contagion of the simulated map had a larger value. However, when these landscape shape metrics were examined by urban density classes, differences were smaller (Fig 9). In general, most values of simulated map indices were similar but slightly smaller than observed map indices, suggested that the model could perform reasonably well when simulating the number, shape, and fragmentation of the urban density classes, especially if its tendency for underestimation was recognized and considered.

### Prediction of urban densification

**Fig 10:**
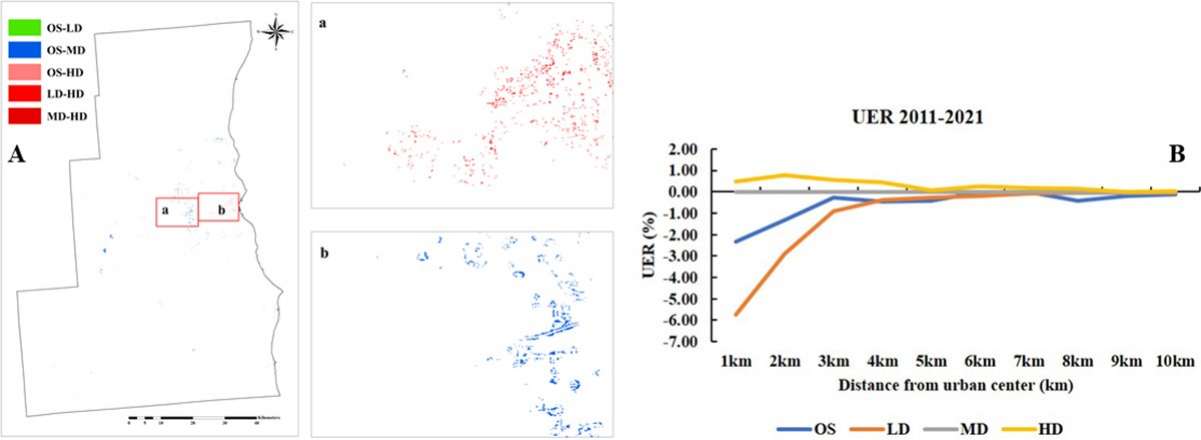
Urban density and Urban Expansion Rate (UER) difference between observed 2011 and simulated 2021. (A) Urban density difference between observed 2011 and simulated 2021. (B) Urban Expansion Rate (UER) difference between observed 2011 and simulated 2021.Labels of XX-YY indicated that observed 2011 was XX and simulated 2021 was YY.

Based on the simulated maps of SEWI in 2016 and 2021, the county of Milwaukee and its surrounding area were still the primary areas for urban densification. Areas predicted to transition to high density were found most commonly in the urban core and were surrounded by predicted medium- and low-density transitions. Many open space and low-density areas were likely to be scattered in locations far from the urban center. The expansion of medium (OS-MD) and high-density areas (OS-HD, LD-HD, and MD-HD) were expected to be characteristic changes between 2011 and 2021 (Fig 10).

Unlike the high absolute values of UER characteristic of 2001 to 2011, the absolute values of UER for 2011 to 2021 were expected to be very low beyond 4 km from the urban center (Fig 10). Within 4 km of the geographic center, high density will likely continue to increase at the expense of open space and low density.

### Implications of urban densification

To realize economic agglomeration advantages, lower density densification, area redevelopment and layout of new areas with higher densities should be included in the urban planning and, if applicable, densification plans [84].

Construction on non-urban areas and open space areas means lower development cost and more “free” expand space than on the higher density areas. However, these “freestyle” urban expansion trends were prone to waste their development potential and generate urban sprawl, congestion and segregation. On account of pressure of population density and urban land supply in the urban core, higher density area presented a characteristic of intensive expansion and fragmentation instead.

These urban patterns were making cities less pleasant and equitable places in which to live.

Intensive and effective urban land use mode was an effective pathway for sustainable urbanization. Promoting the utilization potentiality of lower density, arranging all types of urban density scientifically and rationally will be an effective way to reduce urban land supply pressure especially in the urban core in SEWI. Furthermore, we believed that urban planning, as a solid instrument, combined with realistic financial strategies and policy and legal frameworks, could support the development of quality urban density definitely.

## Discussion

Urban change (urbanization) has dominated land change science for the last several decades. However, there have been very few studies on what some scholars call the urban densification process (urban intensity expansion) despite its importance to the environment and to local economies. The present study contributed to existing knowledge in a comprehensive study by using a rich dataset from the USA and by adapting the well-known Land Transformation Model, namely LTM, which is a free powerful tool for researchers and urban planners[56].

According to the transitions rules obtained from temporal and spatial analysis of urban densification in 2001, 2006, and 2011, we used a reconfigured LTM to predict future urban densification. Based on established evaluation metrics, the reconfigured LTM performed relatively well to simulate urban densification in 2006 and 2011, enabling us to forecast densification in 2016 and 2021. The modelling results revealed that the reconfigured LTM could perform relatively well for most of the urban density changes by considering current urban density and spatial predictor variables such as elevation, slope, and distance to roads, water, and parks.

The results of this study highlighted that the LTM can be modified to incorporate various categories of urban densities (open space (OS), low density (LD), medium density (MD), and high density (HD)) which has provided valuable information for city planners who need to explore the associations between input features of urban densification. We have extended the LTM model and applied it to study urban densification process, using the SEWI-USA area as a case study for the first time. Our results provide researchers working in land change science with important insights into urban densification process modeling. Understanding the driving factors underlying expansion and densification processes was essential for designing policies that support improved land reusing and infilling, and redevelopment.

Despite this success, it would be wise to consider some other socio-economic factors such as the zoning, land price, population density, income levels, and accessibility to local amenities, which could greatly impact the nature of urban densification. Because such socioeconomic variables are always counted by administrative unit that different from predictors (in grid) we used in the paper, we did not include such socioeconomic variables in this paper. Exploration of such additional predictor variables is worthy of future research.

Observed transitions to MD and HD had relatively large percentages of error for both 2006 and 2011, meaning that predicting the changes to these two categories is difficult. Along with urban densification, the fragmentation (ED) and complexity (LSI and PARA_MN) of MD and HD increased intensely (Table 4). Additional insights could likely be gained through the study of more detailed categories of different urban density areas such as residential areas, commercial areas, and industrial areas within high-density or medium-density areas. Additionally, these subcategories could be considered with specific relevant predictor variables.

## Conclusions

According to the research of UN-Habitat, most cities in the world have forfeited agglomeration benefits and generated sprawl, congestion and segregation in the last two decades. The densification strategy, which was an effective tool for improving sustainability of cities has gained much consideration of the public and the research area. This paper documented past urban densification and forecast future densification in southeastern Wisconsin (SEWI) by using a rich dataset from the United States and by adapting the well-known land transformation model (LTM).

Urban densification was a significant phenomenon that often accompanies urbanization more generally. The increasing proportion of lower density areas rather than higher density areas was the main characteristic of the urban densification in SEWI from 2001 to 2011. We believe that urban densification is an important and progressive process along with the urbanization. On account of pressure of population density and urban land supply in the urban core, higher density area presented a characteristic of intensive expansion and fragmentation instead. Another issue need to be addressed along with densification were the increasing runoff and land fragmentation due to the increasing impervious surfaces, the reduced green space and reduced air quality due to growing residential density. We believed that improve the urban land use efficiency and maintain rational densification are both effective pathways for sustainable urbanization.

Multiple goodness-of-fit metrics such as error locations, error quantities, spatial patterns of urban density classes, and model errors demonstrated that the reconfigured LTM performed relatively well to simulate urban densification in 2006 and 2011, enabling us to forecast densification in 2016 and 2021. We found that Milwaukee County and the surrounding area are still the primary areas for urban densification in 2016 and 2021. The expansion of medium (OS-MD) and high-density areas (OS-HD, LD-HD, and MD-HD) were expected to be characteristic changes between 2011 and 2021 which indicated that future urban densification will likely be characterized by higher density continue to increase at the expense of lower densities.

We argue that detailed categories of urban density and specific relevant predictor variables (such as the zoning, land price, population density, income levels, and accessibility to local amenities) were indispensable for densification forecasts. Our study provides researchers working in land change science with important information into urban densification process modeling. The outcome of this model can help planners to identify the current trajectory of urban development, enabling them to make informed decisions to promote planning objectives, which could benefit sustainable urbanization definitely. Indeed, recent calls for coupling land use and climate change forecasts to a variety of ecological models have grown recently and this work represents one form that considers an area of land change that is often ignored. More work is also needed that examines land use functional dimensions across these densities of urban as well [84].

## Supporting information

S1 Dataset The source of data in our paper. (ZIP)
